# Development and validation of nomograms predicting overall and cancer-specific survival for non-metastatic primary malignant bone tumor of spine patients

**DOI:** 10.1038/s41598-023-30509-y

**Published:** 2023-03-01

**Authors:** Yiming Shao, Zhonghao Wang, Xiaoya Shi, Yexin Wang

**Affiliations:** 1 Department of Clinical Medicine, Jining Medical University, No. 133, Hehua Road, Jining, Shandong China; 2 Department of Oncology, Liyuan Hospital, Tongji Medical College, Huazhong University of Science and Technology, 39 Yanhu Avenue, Wuchang District, Wuhan, Hubei Province China; 3 Department of Spine Surgery, Affiliated Hospital of Jining Medical University, 89 Guhuai Road, Jining, Shandong China

**Keywords:** Bone cancer, Cancer epidemiology

## Abstract

At present, no study has established a survival prediction model for non-metastatic primary malignant bone tumors of the spine (PMBS) patients. The clinical features and prognostic limitations of PMBS patients still require further exploration. Data on patients with non-metastatic PBMS from 2004 to 2015 were extracted from the Surveillance, Epidemiology, and End Results (SEER) database. Multivariate regression analysis using Cox, Best-subset and Lasso regression methods was performed to identify the best combination of independent predictors. Then two nomograms were structured based on these factors for overall survival (OS) and cancer-specific survival (CSS). The accuracy and applicability of the nomograms were assessed by area under the curve (AUC) values, calibration curves and decision curve analysis (DCA). Results: The C-index indicated that the nomograms of OS (C‐index 0.753) and CSS (C‐index 0.812) had good discriminative power. The calibration curve displays a great match between the model’s predictions and actual observations. DCA curves show our models for OS (range: 0.09–0.741) and CSS (range: 0.075–0.580) have clinical value within a specific threshold probability range compared with the two extreme cases. Two nomograms and web-based survival calculators based on established clinical characteristics was developed for OS and CSS. These can provide a reference for clinicians to formulate treatment plans for patients.

## Introduction

Primary malignant bone tumors of the spine (PMBS) are relatively rare among all bone tumors, accounting for only 5% or less^1^. The most common PMBS include chordoma, osteosarcoma, chondrosarcoma, and Ewing sarcoma^2^. Local pain is the most common symptom, and it is more common and severe in patients with malignant tumors than in those with benign tumors^1^. Other manifestations are neurological symptoms with spinal cord compression, spinal instability, and pathological fractures^1,3,4^. For PMBS, many cases are unsuitable for total spinal resection due to the complex configuration of the spine^5^. Although intralesional resection of malignant tumors provides functional and pain relief, it results in a high incidence of local recurrence^1^.

Non-metastatic PBMS patients have a better prognosis due to relatively lower malignancy^6^. However, the clinical characteristics and prognostic opportunities of this group of patients remain unclear due to few studies. At present, no research has established a survival prediction model for non-metastatic PMBS patients. Many past studies were limited to small sample cases, or data and analytical methods were relatively old^2,7^. An aggressive multimodal strategy for these tumors is warranted due to their high recurrence rate, functional disease, and limited survival. Advanced modelling methods should be used to build well-applied predictive models based on large populations.

The nomogram is a statistical model that generate a probability of several clinical events for a particular individual^8–11^. Therefore, we aimed to create predictive nomograms and web-based survival calculators based on the SEER database. These tools are used for dynamic prediction of long-term overall survival (OS) and cancer-specific survival (CSS) in patients with non-metastatic PBMS. These results can help specialists develop better clinical strategies.

## Materials and methods

### Study population

Patients diagnosed with nonmetastatic PMBS from 2004 to 2015 were selected from the Surveillance, Epidemiology, and End Results (SEER) database using SEER*stat software (version 8.4.0). The analysis was exempt from medical ethics review and did not require informed consent, as patient-specific information was not included in the SEER database. The patient criteria for inclusion were as follows: (1) histological diagnosis of primary malignant bone tumor in patients between 2004 and 2015. (2) confined to the bony spine: C41.2 (vertebral column) and C41.4 (pelvis, sacrum, coccyx and associated joints). (3) Active follow-up to ensure reliable patient status. We classified patients coded as “distant” as metastatic and those coded as “local” or “regional” as nonmetastatic. After excluding patients with distant metastases and patients with unknown tumor size and surgical information, we included the variables for analysis as follows: age, sex, race, marital status, year of diagnosis, tumor size(mm), laterality, grade, radiation, chemotherapy, surgery, surgery combined with radiation, historic stage, histological type, cause of death, vital status and survival months. The end events were defined as all-cause and specific deaths. Therefore, the evaluation metrics consist of OS time, CSS time, OS state and CSS state.

### Statistical analysis

Pearson’s chi-square analysis was used to evaluate different clinical characteristics between different treatment regimens. All statistical tests were two-sided, and a *P* value < 0.05 was considered statistically significant. The log-rank test was applied to test the differences between groups. Determination of the optimal cut-off point for tumor size was performed using X-tile software (version 3.6.1)^12^. All statistical analyses were performed using R4.1.3 software (The R Project for Statistical Computing, http://www.r-project.org).

#### Construction of the nomogram

All patients were divided into a validation group and a training group at a ratio of 3:7 using R programming language software. Multivariate regression analysis using Cox, Best-subset and Lasso regression three methods was performed to compare and find the best combination of variables to identify independent predictors of survival. The forest plot shows the effect of different variables on survival outcomes results. Then prognostic prediction nomograms for OS and CSS were established.

#### Evaluation of the performance

The accuracy of the nomogram was assessed by area under the curve (AUC) values and calibration curves, and the clinical benefits was assessed by decision curve analysis (DCA). A standard curve was generated using the bootstrap method. The cohort was tested 1000 times for internal validation.

#### Web-based survival rate calculator

Based on the nomogram, two web-based survival rate calculators were further created for OS and CSS.

#### Survival analysis risk stratification

After calculating the total score of all patients, according to the best cut-off value supplied by X-tile software, all patients were divided into three subgroups: low, medium and high risk. Finally, survival differences between subgroups were compared separately using Kaplan‒Meier survival analysis.

### Ethics approval and consent to participate

The study was conducted according to the guidelines of the Declaration of Helsinki, and the ethical approval for using SEER database and retrospective data is waived.

## Results

### Patient characteristics

A total of 1137 patients were included in this study and subjected to OS and CSS analysis, which were randomized into training (n = 795) and validation groups (n = 342) (Table 1). Among all non-metastatic PMBS patients, males (654, 57.5%) were more likely to be male than females (483, 42.5%) in terms of sex, and white people (974, 85.7%) were the majority in terms of racial information. Regarding histological types, chondrosarcoma (394, 34.7%) had the highest proportion, followed by chordoma (320, 28.1%), Ewing sarcoma (156, 13.7%) and osteosarcoma (145, 12.8%), which had similar distributions, and malignant giant cell tumor of bone (15, 1.3%) had the lowest proportion. Among the tumor sizes, 50–129 mm (659, 58.0%) was the most common. Regional (671, 59.0%) is higher than Localized (466, 41.0%) in the historic stage. Most patients had surgery (825, 72.6%), and only a few opted for radiation (406, 35.7%) or chemotherapy (352, 31.0%). The difference between the training and validation groups was not statistically significant (P > 0.05).

**Table 1:** Demographics and clinical characteristics of patients.

Variables	All (N = 1137)	Test (N = 342)	Train (N = 795)	*P* value
Age				
< 30	280 (24.6%)	80 (23.4%)	200 (25.2%)	0.783
≥ 60	416 (36.6%)	125 (36.5%)	291 (36.6%)	
30–59	441 (38.8%)	137 (40.1%)	304 (38.2%)	
Sex				
Female	483 (42.5%)	140 (40.9%)	343 (43.1%)	0.513
Male	654 (57.5%)	202 (59.1%)	452 (56.9%)	
Race				
Black	77 (6.8%)	21 (6.1%)	56 (7.0%)	0.135
Other	86 (7.6%)	34 (9.9%)	52 (6.5%)	
White	974 (85.7%)	287 (83.9%)	687 (86.4%)	
Marital status				
Married	550 (48.4%)	167 (48.8%)	383 (48.2%)	0.846
Unmarried	587 (51.6%)	175 (51.2%)	412 (51.8%)	
Year of diagnosis				
2004–2009	491 (43.2%)	152 (44.4%)	339 (42.6%)	0.602
2010–2015	646 (56.8%)	190 (55.6%)	456 (57.4%)	
Tumor size (mm)				
< 50	284 (25.0%)	80 (23.4%)	204 (25.7%)	0.096
> 129	194 (17.1%)	71 (20.8%)	123 (15.5%)	
50–129	659 (58.0%)	191 (55.8%)	468 (58.9%)	
Laterality				
One site	1115 (98.1%)	335 (98.0%)	780 (98.1%)	0.818
Paired site	22 (1.9%)	7 (2.0%)	15 (1.9%)	
Grade				
I	140 (12.3%)	43 (12.6%)	97 (12.2%)	0.446
II	192 (16.9%)	57 (16.7%)	135 (17.0%)	
III	123 (10.8%)	46 (13.5%)	77 (9.7%)	
IV	166 (14.6%)	48 (14.0%)	118 (14.8%)	
Unknown	516 (45.4%)	148 (43.3%)	368 (46.3%)	
Radiation				
No	731 (64.3%)	223 (65.2%)	508 (63.9%)	0.686
Yes	406 (35.7%)	119 (34.8%)	287 (36.1%)	
Chemotherapy				
No/Unknown	785 (69.0%)	242 (70.8%)	543 (68.3%)	0.442
Yes	352 (31.0%)	100 (29.2%)	252 (31.7%)	
Surgery				
No	312 (27.4%)	88 (25.7%)	224 (28.2%)	0.426
Yes	825 (72.6%)	254 (74.3%)	571 (71.8%)	
Surgery with radiation				
No	899 (79.1%)	274 (80.1%)	625 (78.6%)	0.633
Yes	238 (20.9%)	68 (19.9%)	170 (21.4%)	
Historic stage				
Localized	466 (41.0%)	136 (39.8%)	330 (41.5%)	0.599
Regional	671 (59.0%)	206 (60.2%)	465 (58.5%)	
Histology				
Chondrosarcoma	394 (34.7%)	123 (36.0%)	271 (34.1%)	0.727
Chordoma	320 (28.1%)	91 (26.6%)	229 (28.8%)	
Ewing sarcoma	156 (13.7%)	45 (13.2%)	111 (14.0%)	
Giant cell tumor of bone	15 (1.3%)	5 (1.5%)	10 (1.3%)	
Osteosarcoma	145 (12.8%)	40 (11.7%)	105 (13.2%)	
Other	107 (9.4%)	38 (11.1%)	69 (8.7%)	

### Nomogram variable screening

To obtain the best combination of variables, we used Cox, Best-subset and Lasso regression models to perform multivariate stepwise regression analysis on the clinical characteristics in the training group. Then we used AUC values to compare the three models in the 1-year, 3-year, 5-year and 10-year survival rates of OS and CSS and found that the model screened by Cox outperformed all the others in each situation (Fig. S1). By multivariate Cox stepwise regression, the AUC values for 1-year, 3-year, 5-year and 10-year OS were 0.809 (95% CI = 0.749–0.869), 0.799 (95% CI = 0.744–0.854), 0.790 (95% CI = 0.741–0.838) and 0.779 (95% CI = 0.720–0.837), and for 1-year, 3-year, 5-year and 10-year CSS were 0.831 (95% CI = 0.760–0.902), 0.856 (95% CI = 0.792–0.921), 0.842 (95% CI = 0.790–0.903) and 0.822 (95% CI = 0.756–0.888), respectively.

**Figure 1:**
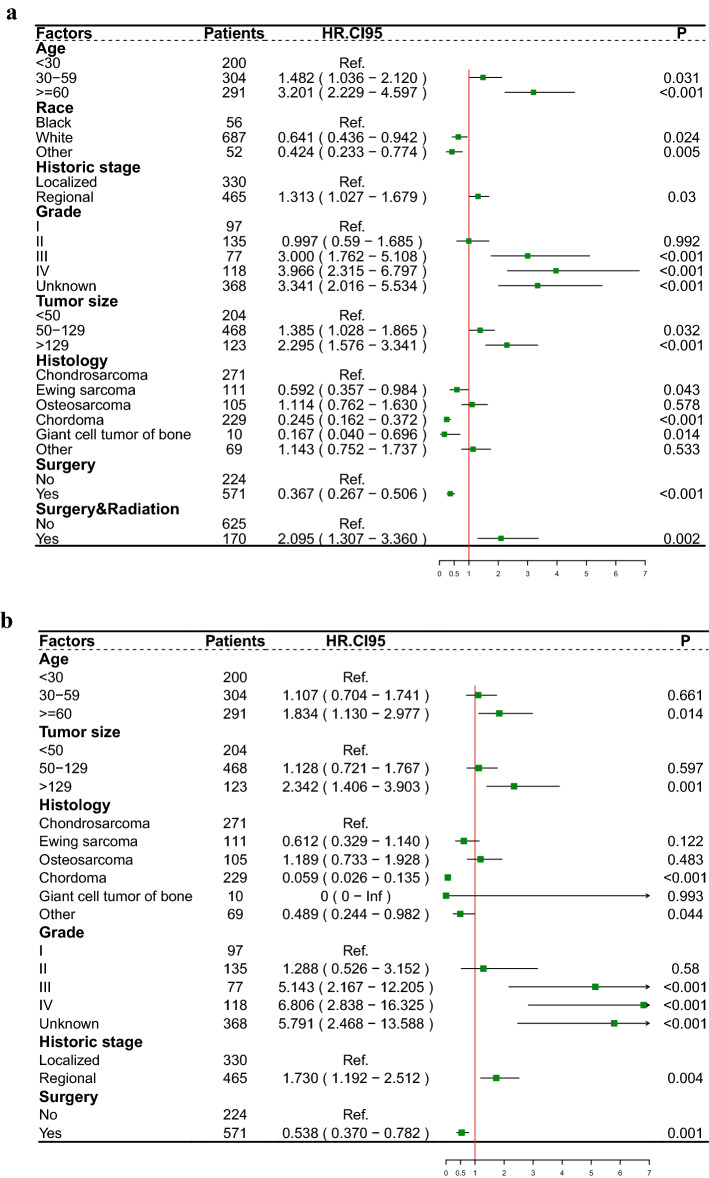
Forest plot depicting the effect of different prognostic factors on overall survival (OS) (**a**) and cancer-specific survival (CSS) (**b**).

Ultimately, age, race, historic stage, histology, tumor size, grade, surgery, and surgery combined with radiation were identified as independent prognostic factors for OS (Fig. 1a). Age, historic stage, histology, tumor size, grade and surgery were ascertained as separate prognostic factors for CSS (Fig. 1b). We performed a Kaplan‒Meier survival analysis of the overall population based on separate prognostic risk factors for OS (Fig. S2) and CSS (Fig. S3). The results revealed that age < 30 years (P < 0.001), other races (P = 0.035), Grade I and II (P < 0.001), chordoma (P < 0.001), localized stage (P < 0.001), tumor size < 50 mm (P < 0.001), surgery (P < 0.001) and surgery combined with radiation (P = 0.005) had better OS, and age < 30 years (P < 0.001), Grade I and II(P < 0.001), chordoma and malignant giant cell tumor of bone (P < 0.001), localized stage (P < 0.001), tumor size < 50 mm (P < 0.001) and surgery (P < 0.001) had better CSS.

### Nomogram construction and validation

**Figure 2:**
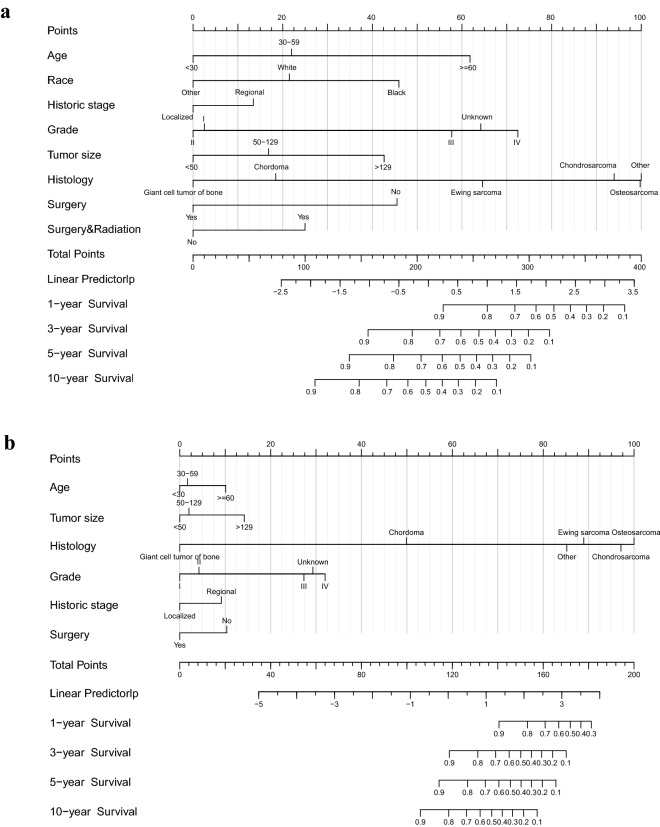
The prognostic nomograms for OS (**a**) and CSS (**b**).

We constructed nomograms of the training group in non-metastatic PMBS patients based on the screened variables to predict 1-year, 3-year, 5-year and 10-year OS and CSS in non-metastatic PMBS patients (Fig. 2). The C‐index value was 0.753 (95% CI = 0.726–0.780, P < 0.001) for OS and 0.812 (95% CI = 0.782–0.841, P < 0.001) for CSS. We can calculate the patient's total score to assess survival rates, for example, a 65-year-old white patient with regional osteosarcoma and with Grade II, 65 mm tumor size, who underwent surgery only. Consequently, probability OS at 1-year, 3-year, 5-year and 10-year is 0.920 (95% CI = 0.870–0.960), 0.730 (95% CI = 0.620–0.870), 0.650 (95% CI = 0.510–0.820) and 0.460 (95% CI = 0.303–0.710), probability CSS at 1-year, 3-year, 5-year and 10-year is 0.950 (95% CI = 0.920–0.990), 0.850 (95% CI = 0.750–0.960), 0.810 (95% CI = 0.690–0.950) and 0.710 (95% CI = 0.550–0.920), respectively.

We used the test group to validate the nomogram. In the test group, the AUCs of models forecasting 1-year, 3-year, 5-year and 10-year OS (Fig. S4) were 0.857 (95% CI = 0.799–0.915), 0.857 (95% CI = 0.779–0.936), 0.837 (95% CI = 0.763–0.911) and 0.814 (95% CI = 0.744–0.885). In CSS, the AUCs of 1-year, 3-year, 5-year and 10-year (Fig. S4) were 0.871 (95% CI = 0.789–0.952), 0.877 (95% CI = 0.781–0.974), 0.865 (95% CI = 0.776–0.954) and 0.853 (95% CI = 0.762–0.944), respectively. The results show that both models perform well in forecasting OS and CSS, with AUC values > 0.814 in all test groups, either OS or CSS, indicating good discrimination of nomograms. Calibration curves for nomograms showed a high degree of agreement, in the training and validation groups, between observed and predicted survival probabilities (Fig. S5). We simultaneously studied and validated the clinical value of the nomogram. Comparing the clinical benefits of our models with the validation group, the DCA curves showed that the clinical utility of nomogram-based OS (Fig. S6) and CSS (Fig. S7) at 1 year, 3 years, 5 years and 10 years was almost identical to the actual observations in the two groups. In the DCA curve, the Y-axis measures the net benefit, and the X-axis is the threshold probability of needing intervention^13,14^. The area under the curve indicates the clinical utility of the model, and the farther the model curve is from the ‘no treatment’ or ‘all treatment’ line, the better the clinical value. Our models for OS (range: 0.09–0.741) and CSS (range: 0.075–0.580) have clinical value within a specific threshold probability range compared with the two extreme cases. In conclusion, the non-metastatic PMBS nomogram has considerable discriminative and calibration power and exhibits clinical benefits consistent with that observed, implying that it has better implications for clinical implementation.

### Web-Based survival rate calculator

**Figure 3:**
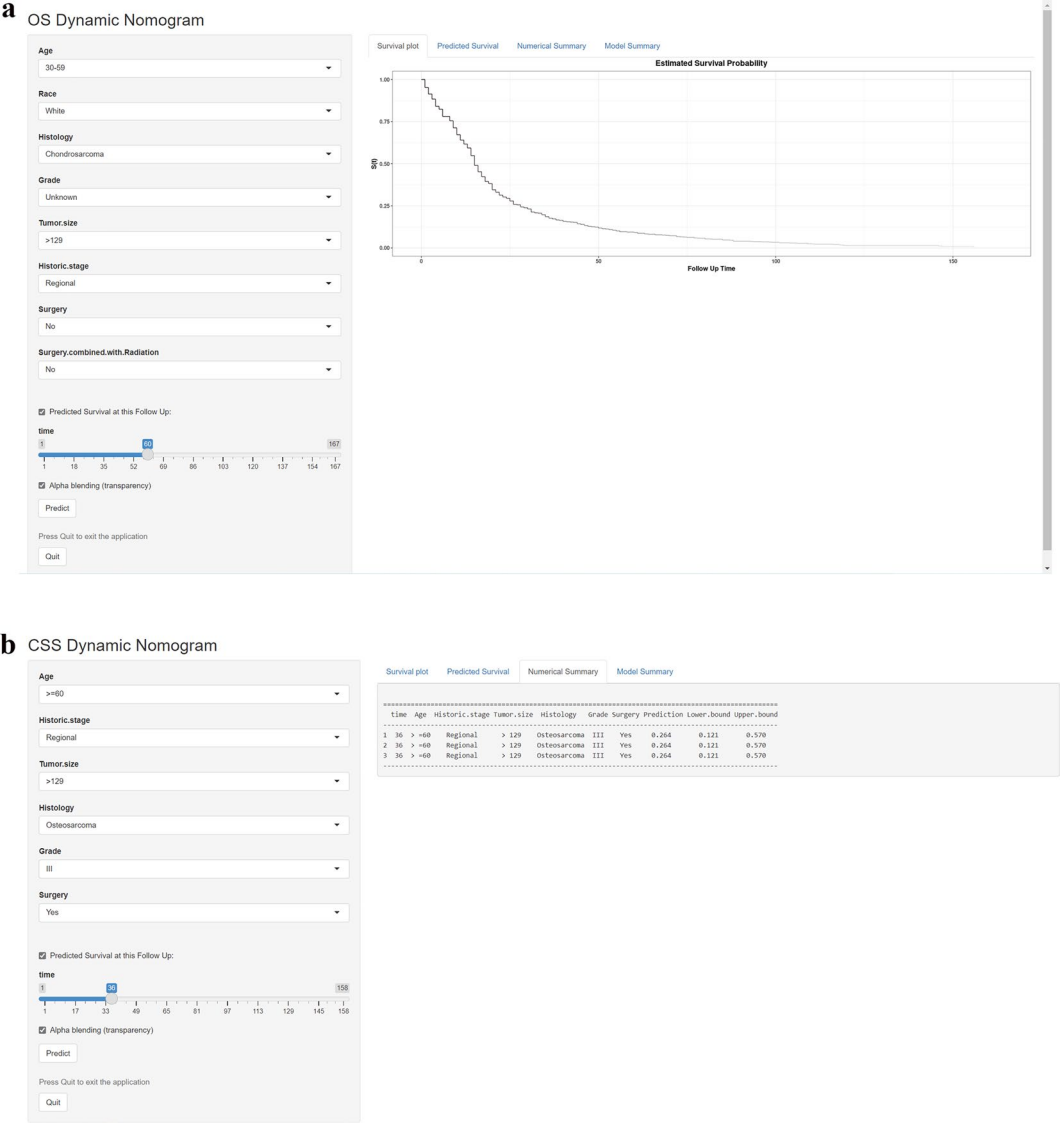
Web-based survival rate calculator used to predict non-metastatic PMBS patient OS (**a**) and CSS (**b**).

To more intuitively use the nomogram for clinical policy-making, we besides established two network-based survival calculators for figuring OS (https://rocksyy.shinyapps.io/OSDynNomapp/) and CSS (https://rocksyy.shinyapps.io/CSSDynNomapp/) in patients with non-metastatic PMBS (Fig. 3). Just enter the corresponding patient information on the left side of the net surface to report the survival curve and evaluate survival probability.

### Survival analysis by risk stratification

**Figure 4:**
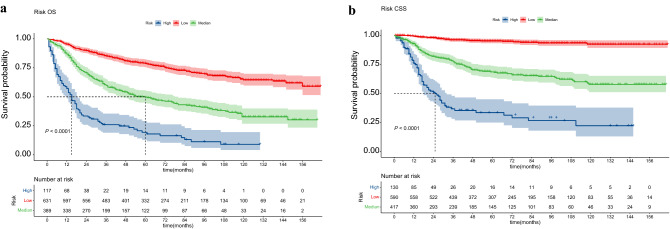
Risk subgroup analysis of OS (**a**) and CSS (**b**) using the Kaplan‒Meier method.

In addition, we calculated a risk score for each non-metastatic PMBS patient in the overall population based on a nomogram model. We divided the enrolled patients into three risk subgroups according to the optimal cut-off value provided by X-tile software. The risk groups for OS involved the low-risk group (n = 631, score 12.52–178.16 points), intermediate-risk group (n = 389, score 178.68–259.49 points), and high-risk group (n = 117, score 260.41–369.52 points). The risk groups for CSS included the low-risk group (n = 590, score 37.85–122.23 points), intermediate-risk group (n = 417, score 122.32–149.78 points), and high-risk group (n = 130, score 149.92–179.78 points). We performed survival analysis for the three subgroups using the Kaplan‒Meier method, and each risk subgroup represented a different prognosis (Fig. 4). The results revealed that both OS (P < 0.001) and CSS (P < 0.001) in the three subgroups were precisely separated. The prognosis of patients with low-risk scores was better than those with high-risk scores, reflecting that the risk stratification built based on the nomogram has available prophetic value for the prognosis of patients with non-metastatic PMBS.

## Discussion

Primary malignancies of the spine are the rarest types of tumors in the spine, and only 10% of all bone and soft tissue sarcomas are related to the spine^6^. Surgery and radiation therapy may impair the normal function of the spinal cord and cause neurological deficits due to the complex structure of the spine^15^. Therefore, treatment options for patients with non-metastatic PMBS are more challenging, and these have not been fully explored.

The results of this study showed that overall survival and cancer-specific survival were 88.5% and 94.4% at 1 year, 70.5% and 83.4% at 3 years, 62.9% and 79.4% at 5 years, and 48.0% and 72.6% at 10 years of non-metastatic PMBS, respectively. In this study, we focused on the four most common bone tumors of non-metastatic PMBS: chondrosarcoma, chordoma, Ewing sarcoma, and osteosarcoma^16^. According to the SEER database, chondrosarcoma is the most common, accounting for 34.7% of all patients, followed by chordoma (28.1%), Ewing sarcoma (13.7%) and osteosarcoma (12.8%) in similar numbers of patients.

Our study found that older age (> 60 years old), tumor size > 129 mm, and Grade III and IV had relatively poor OS and CSS. While surgery combined with radiotherapy is an unfavorable factor for OS, surgery can significantly improve both OS and CSS in non-metastatic PMBS patients. Histological type was the most substantial independent prognostic factor, with the lowest risk for chordoma and the highest risk for osteosarcoma, whether OS or CSS.

Age is an independent prognostic factor for non-metastatic PMBS, regardless of OS or CSS, and the age distribution of patients is generally related to histological type^17^. Unlike previous studies involving metastatic patients^18–21^, in our study of non-metastatic patients, the patients were relatively older, and the vast majority of patients (76.3%) were older than 30 years. In addition, Ewing sarcoma patients are relatively young, with 81.4% of patients under 30. Correspondingly, chondrosarcoma and chordoma patients were older, with only 11.2% and 5.9% of patients younger than 30 years old, respectively. The reason for the difference is that our study only included non-metastatic patients. We found that with increasing age, patient survival was significantly lower, which agrees with previous studies^22,23^. Older age is a painful factor for both OS and CSS, which may be because of older patients’ poorer physical status or other underlying diseases. So, they inability to tolerate aggressive treatment, and excessive posttreatment complications such as unintentional injury caused by spinal instability, which significantly reduces survival in older patients^24,25^. It should be noted that the impact of ageing on OS is considerably higher than the impact on CSS. This may be due to aging organs and weakened immunity in elderly patients. These conditions lead to an increased likelihood of dying from underlying disease or other systemic disease. So, they are more difficult to tolerate surgery or other adjuvant treatments. Because of the unique position of the spine, accidents caused by spinal instability are unavoidable^26^. These factors reduce the probability of death attributable to non-metastatic PMBS.

Gokaslan et al.^27^ suggested that increased tumor size and infiltration may make tumors more difficult for surgical resection and adjuvant therapy. This is highly consistent with our findings that patients’ OS and CSS decreased significantly with increasing tumor size. Patients with historic stage described as ‘regional’ are at higher risk than those with ‘localized’. This may be related to the fact that it is difficult to obtain sufficient margins due to the large size and deep infiltration, and it is more difficult to completely remove the tumor tissue^28^.

Surgical intervention is widely accepted as the standard treatment strategy for non-metastatic PMBS. Surgical treatment has many profits, such as tumor resection, pain relief and spinal cord decompression^1,29,30^. In a previous study, Mukherjee et al. found that patients with osteosarcoma, chondrosarcoma, Ewing sarcoma, and chordoma who underwent surgery had prolonged survival compared with patients who received medical therapy alone^2^. In this study, the vast majority of patients with chondrosarcoma (85.5%), chordoma (77.2%), osteosarcoma (73.1%), and a minority of patients with Ewing sarcoma (46.2%) underwent surgery, and surgical treatment had an apparent positive effect on both OS and CSS. The therapeutic effect of surgery is related to the method of resection and the condition of the incision margin^23,31^. En bloc tumor resection with wide margins (R0), recommended by many spine surgeons, provides long-term disease control in most non-metastatic PMBS compared to subtotal resection^32,33^. Unfortunately, the SEER database does not contain specific surgical modality information, which limits our study.

This study built a predictive model and a survival calculator in network for non-metastatic PMBS patients. Internal validation showed that the predictive model performed well in discriminating patient outcomes at 1, 3, 5, and 10 years. The nomogram can provide a reference for clinicians to predict patient survival and formulate treatment plans. Nomogram-based risk stratification helps us to intuitively determine the risk level of patients.

It must be acknowledged that our study has some limitations. First, the SEER database, which is the source of the research data, does not contain relevant information such as patients’ surgical methods, chemotherapy regimens, and gene mutations, which makes it impossible for us to analyze the impact of these potential factors on the results. In addition, retrospective studies naturally have their limitations compared to prospective studies. Finally, due to the rarity of non-metastatic PMBS patients, no external validation of the model was performed. The above problems are all directions that we need to improve, and strive to collect expected data and develop more rigorous nomograms in the future.

## Conclusion

A nomogram based on established clinical characteristics was developed that can provide a reference for clinicians to predict survival and formulate treatment plans for patients with non-metastatic PBMS. In addition, two web-based survival calculators have been established. These tools can promote personalized survival assessments for this population.

## Supplementary Information

Supplementary Figure S1.

Supplementary Figure S2.

Supplementary Figure S3.

Supplementary Figure S4.

Supplementary Figure S5.

Supplementary Figure S6.

Supplementary Figure S7.

## Data Availability

All data are available at SEER database (https://seer.cancer.gov/). The data presented in this study are available in this article (and supplementary material).

## Abbreviations

SEER: Surveillance, Epidemiology, and End Results
OS: Overall survival
CI: Confidence interval
HR: Hazard ratio
C-index: The concordance index
ROC: Receiver operating characteristic curve
AUC: The receiver operating characteristic curve
DCA: Determination curve analysis
RT: Radiotherapy
