# Supplementary material for: Development and validation of nomograms predicting overall and cancer-specific survival for non-metastatic primary malignant bone tumor of spine patients
Source: Sci Rep. 2023 Mar 1;13:3503. doi: 10.1038/s41598-023-30509-y (PMC9977926; doi:10.1038/s41598-023-30509-y)
Supplement: Supplementary file 1 — Supplementary Figure S1. [file 41598_2023_30509_MOESM1_ESM.docx]

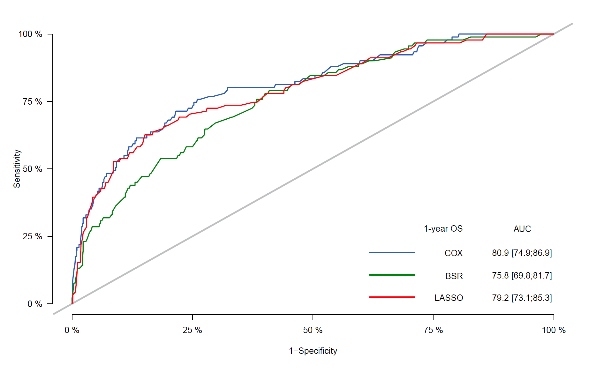

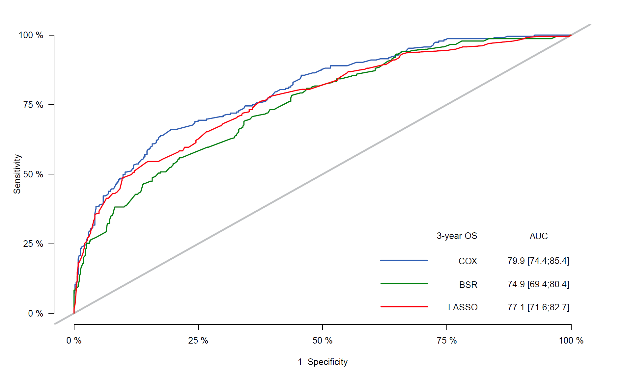


a b


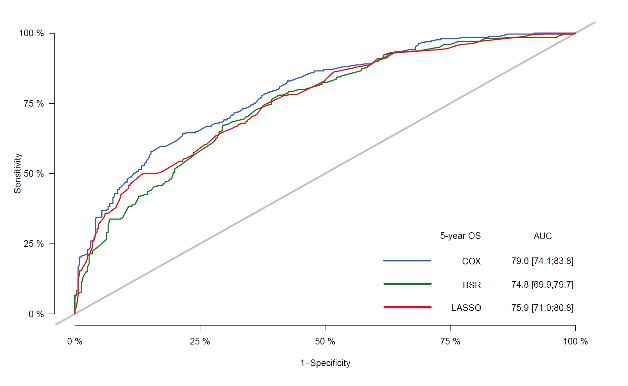

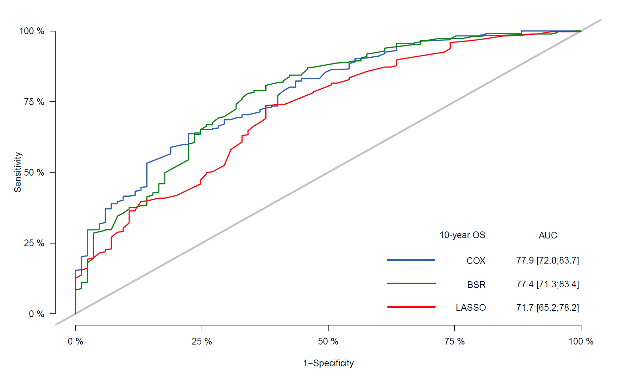


c d


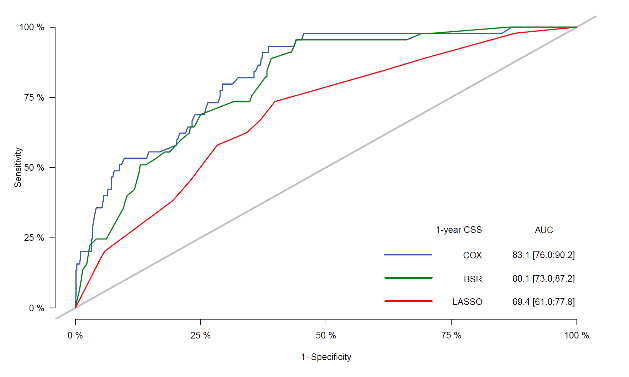

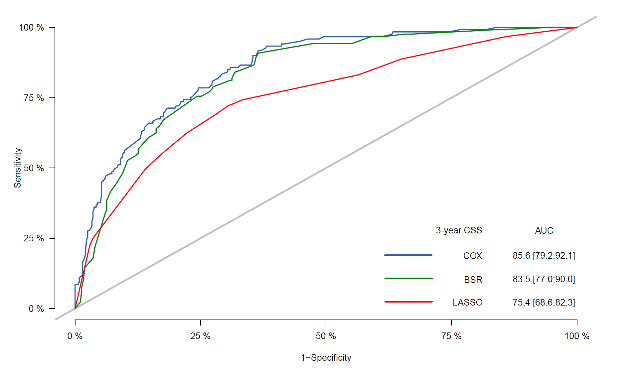


e f


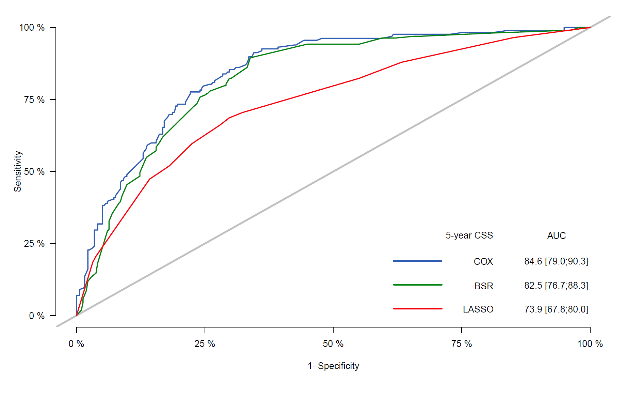

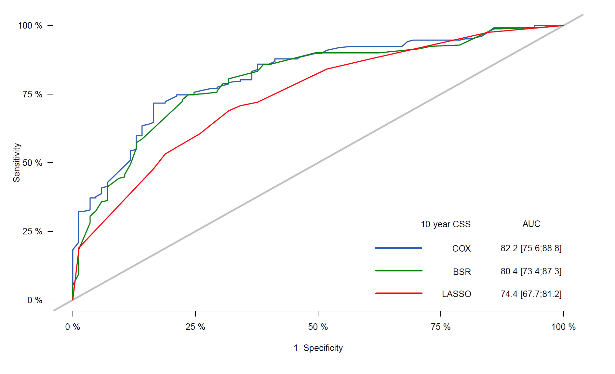


g h

Figure S1 ROC curves of Cox, Best-subset and Lasso regression for 1-year (a), 3-year (b), 5-year (c) and 10-year (d) OS prediction and for 1-year (e), 3-year (f), 5-year (g) and 10-year (h) CSS prediction.
