# Supplementary material for: Development and validation of nomograms predicting overall and cancer-specific survival for non-metastatic primary malignant bone tumor of spine patients
Source: Sci Rep. 2023 Mar 1;13:3503. doi: 10.1038/s41598-023-30509-y (PMC9977926; doi:10.1038/s41598-023-30509-y)
Supplement: Supplementary file 2 — Supplementary Figure S2. [file 41598_2023_30509_MOESM2_ESM.docx]

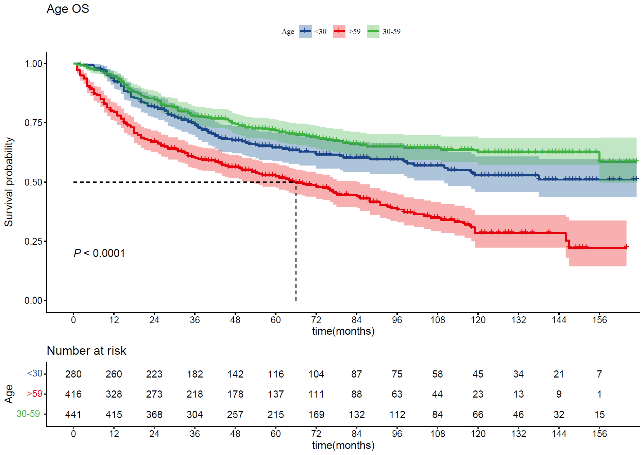

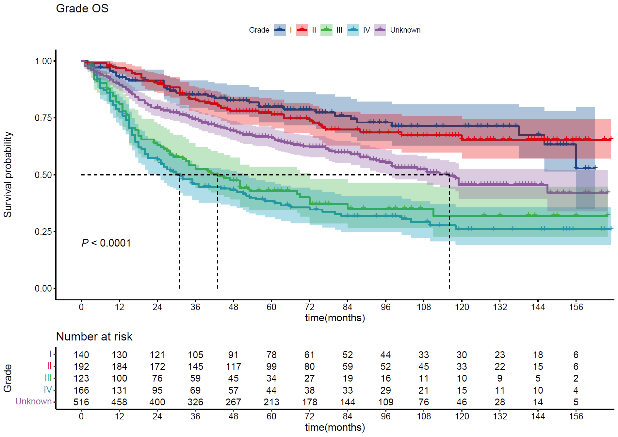


a b


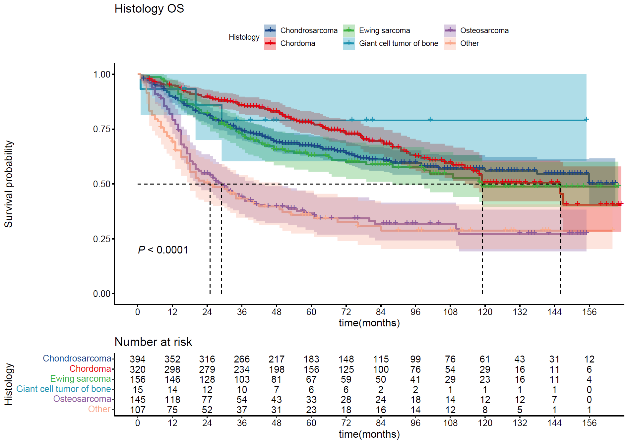

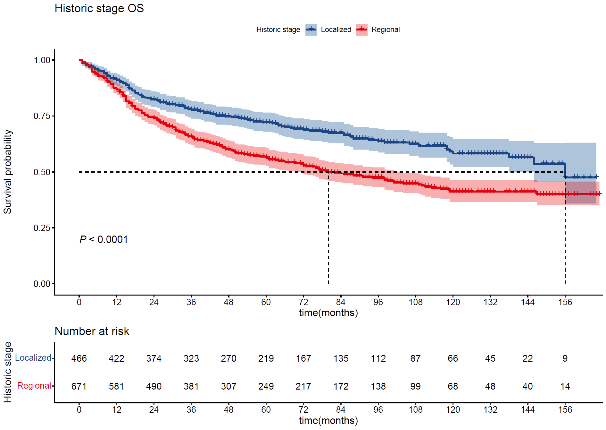


c d


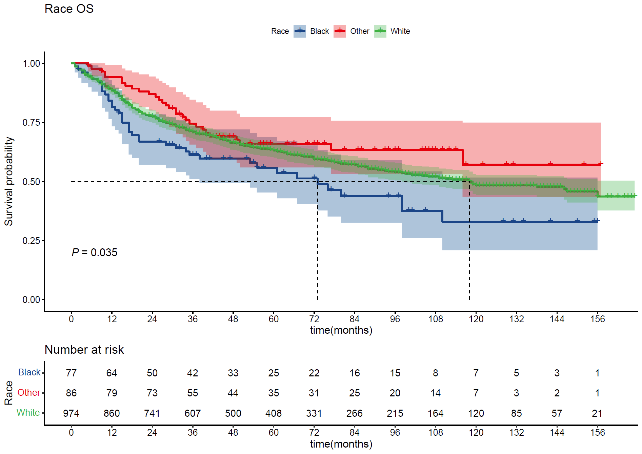

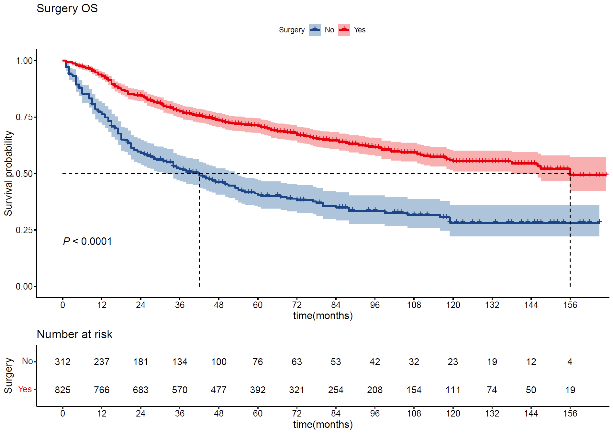


e f


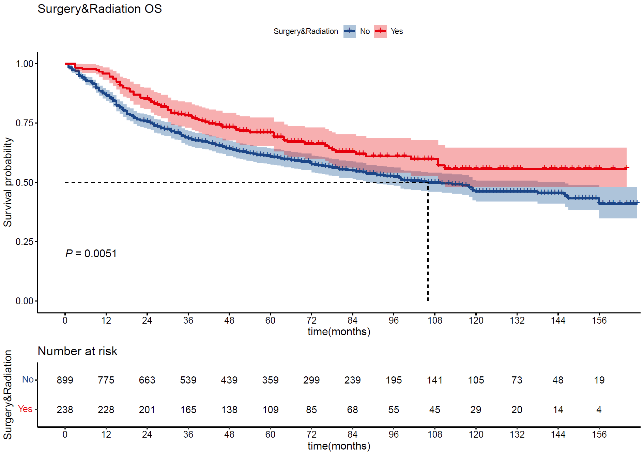

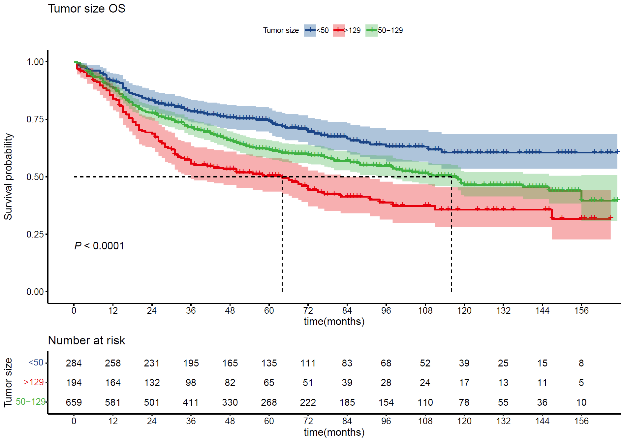


g h

Figure S2 Kaplan–Meier survival analysis for OS. The Kaplan–Meier survival curves of age (**a**, *P* < 0.001), Grade (**b**, *P* < 0.001), histology (**c**, *P* < 0.001), historic stage (**d**, *P* < 0.001), race (**e**, *P* = 0.035), surgery (**f**, *P* < 0.001), surgery combined with radiation (**g**, *P* = 0.005) and tumor size (**h**, *P* < 0.001) for OS. Tumor size in millimeters.
