# Supplementary material for: Development and validation of nomograms predicting overall and cancer-specific survival for non-metastatic primary malignant bone tumor of spine patients
Source: Sci Rep. 2023 Mar 1;13:3503. doi: 10.1038/s41598-023-30509-y (PMC9977926; doi:10.1038/s41598-023-30509-y)
Supplement: Supplementary file 3 — Supplementary Figure S3. [file 41598_2023_30509_MOESM3_ESM.docx]

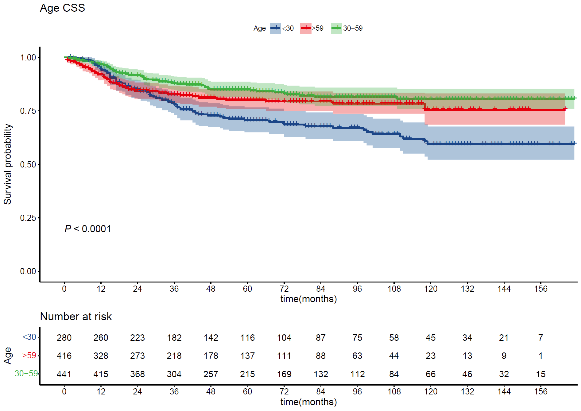

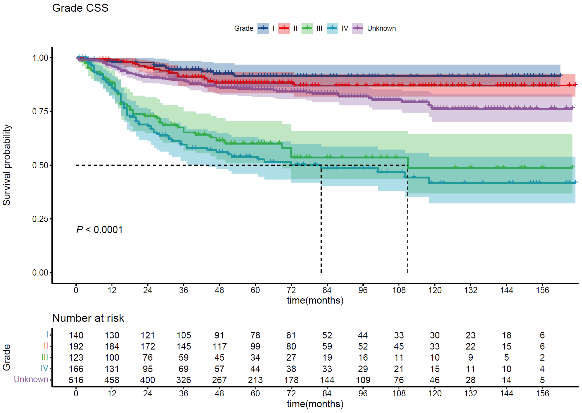


a b


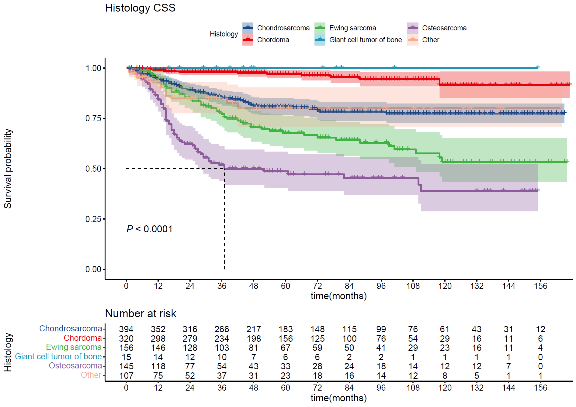

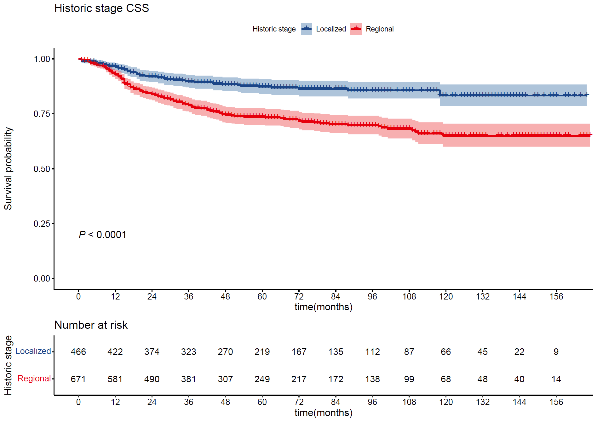


c d


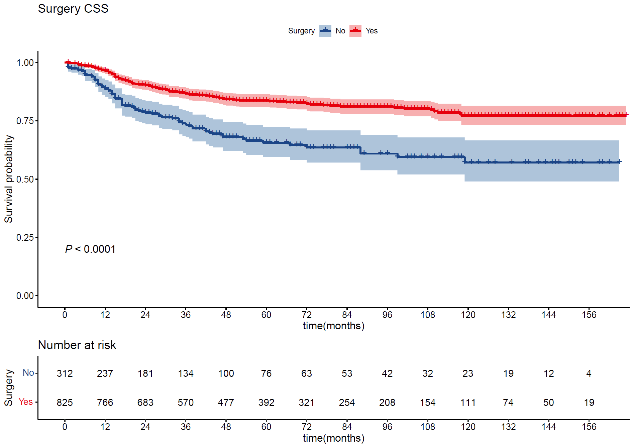

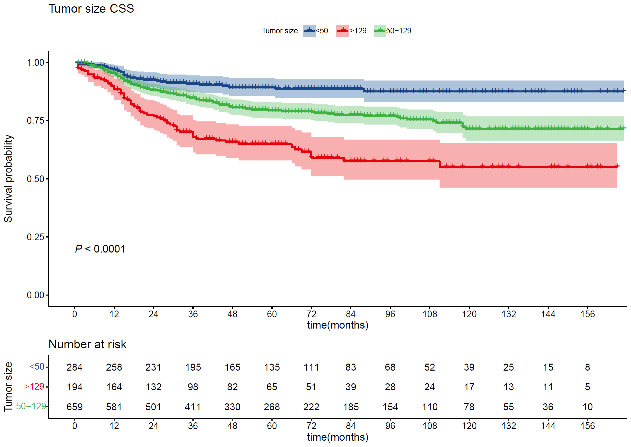


e f

Figure S3 Kaplan–Meier survival analysis for CSS. The Kaplan–Meier survival curves of age (**a**, *P* < 0.001), Grade (**b**, *P* < 0.001), histology (**c**, *P* < 0.001), historic stage (**d**, *P* < 0.001), surgery (**e**, *P* < 0.001) and tumor size (**f**, *P* < 0.001) for CSS. Tumor size in millimeters.
