# Supplementary material for: Development and validation of nomograms predicting overall and cancer-specific survival for non-metastatic primary malignant bone tumor of spine patients
Source: Sci Rep. 2023 Mar 1;13:3503. doi: 10.1038/s41598-023-30509-y (PMC9977926; doi:10.1038/s41598-023-30509-y)
Supplement: Supplementary file 5 — Supplementary Figure S5. [file 41598_2023_30509_MOESM5_ESM.docx]

a b

c d

e f

g h

Figure S5 Comparison of calibration curves of the training group and test group based on the nomogram for 1-year (a), 3-year (b), 5-year (c) and 10-year (d) OS and for 1-year (e), 3-year (f), 5-year (g) and 10-year (h) CSS. The red line represents the test group and the blue line represents the training group. Plots showed that actual survival was closely related to predicted survival.
