# Supplementary material for: Development and validation of nomograms predicting overall and cancer-specific survival for non-metastatic primary malignant bone tumor of spine patients
Source: Sci Rep. 2023 Mar 1;13:3503. doi: 10.1038/s41598-023-30509-y (PMC9977926; doi:10.1038/s41598-023-30509-y)
Supplement: Supplementary file 6 — Supplementary Figure S6. [file 41598_2023_30509_MOESM6_ESM.docx]

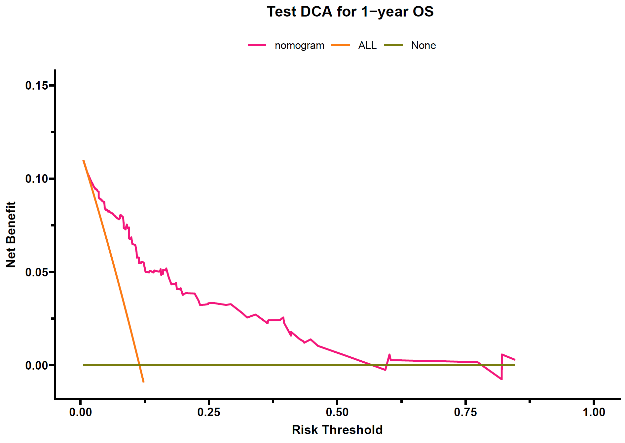

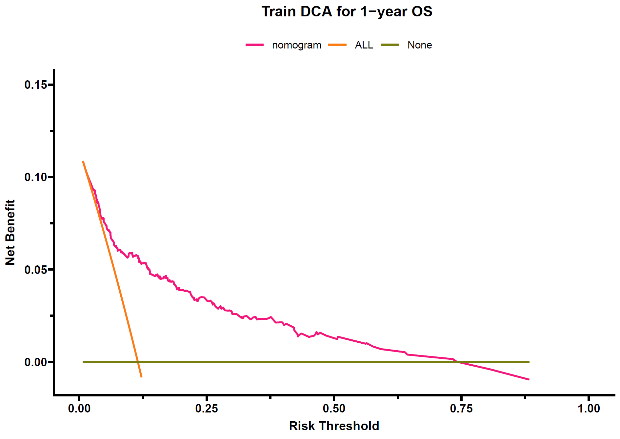


a b


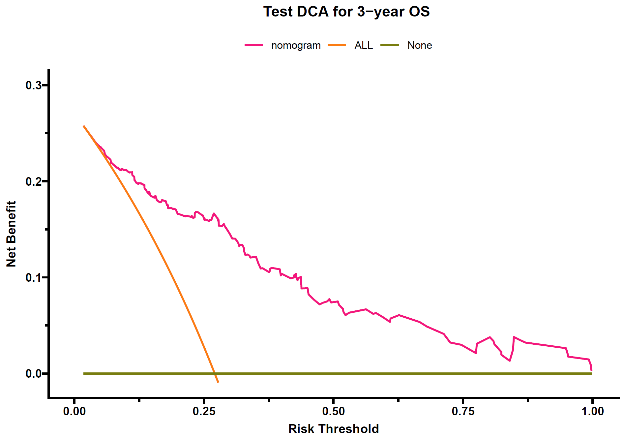

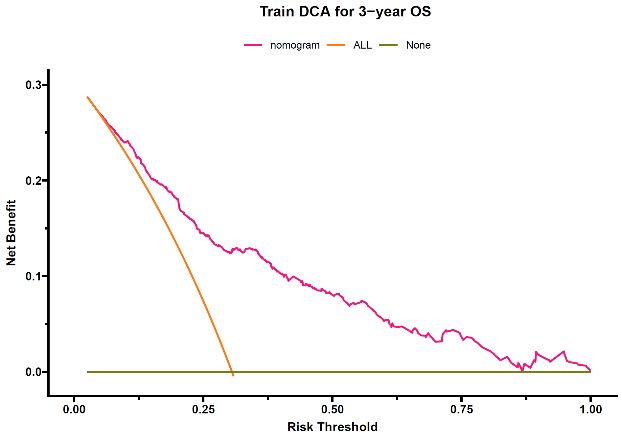


c d


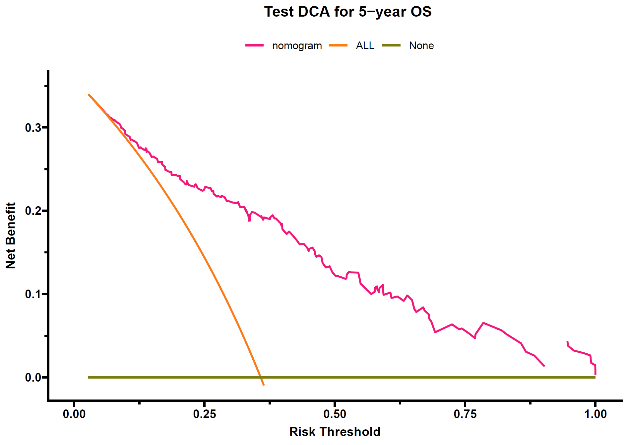

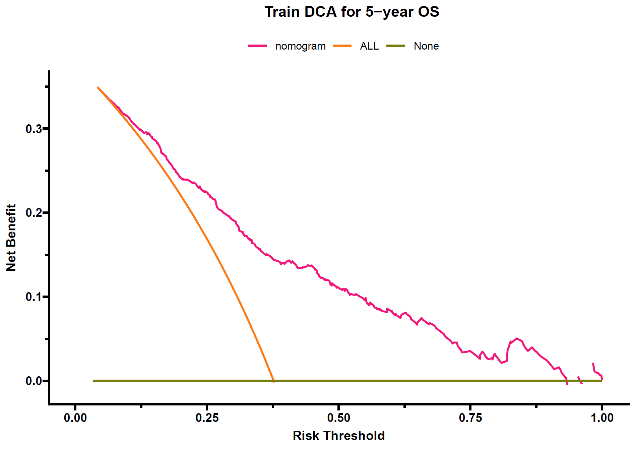


e f


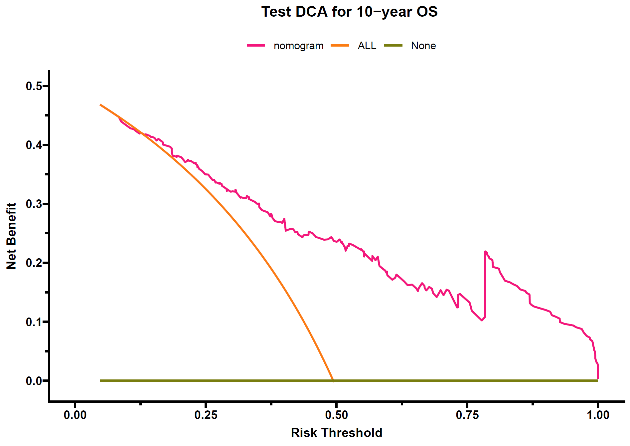

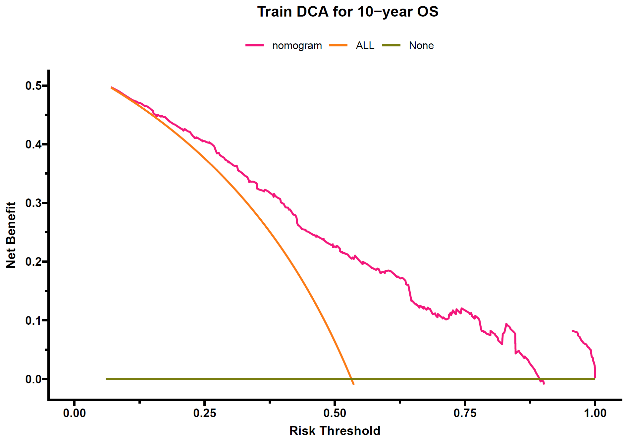


g h

Figure S6 Comparison of DCA curves for 1-year (a), 3-year (c), 5-year (e) and 10-year (g) OS in the test group and for 1-year (b), 3-year (d), 5-year (f) and 10-year (h) OS in the training group based on the nomogram.
